# Tooth agenesis in German orthodontic patients with non-syndromic craniofacial disorder: a retrospective evaluation of panoramic radiographs

**DOI:** 10.1007/s00784-022-04538-2

**Published:** 2022-05-26

**Authors:** C. Weise, M. Lehmann, M. C. Schulz, S. Reinert, B. Koos, H. Weise

**Affiliations:** 1 Department of Orthodontics, University Hospital Tübingen, Osianderstr. 2-8, 72076 Tubingen, Germany; 2 Department of Oral and Maxillofacial Surgery, University Hospital Tübingen, Osianderstr. 2-8, 72076 Tubingen, Germany

**Keywords:** Agenesis, Bilateral cleft lip and palate, Robin sequence, Tooth agenesis code, Unilateral cleft lip and palate

## Abstract

**Objectives:**

The study objective was to evaluate the tooth agenesis in German orthodontic patients with non-syndromic cleft lip and/or palate and Robin sequence compared to a control group without craniofacial disorder.

**Materials/methods:**

A total of 108 panoramic radiographs were examined using the binary system of Tooth Agenesis Code (TAC) (excluding the third molar). Patients were divided into the craniofacial disorder group 1 (*n* = 43) and the healthy control group 2 (*n* = 65). Parameters such as skeletal class malformation, sex, localization of the cleft, craniofacial disorder, and interobserver reliability were assessed.

**Results:**

Permanent tooth agenesis was observed in 44% of group 1 and 14% in group 2 with a statistically significant higher prevalence (*p* = 0.00162 (*χ*^2^)). Fourteen different TAC patterns were observed in group 1, ten of these occurring only once in separate patients. The distribution of the TAC codes in group 2 showed nine different possibilities of TAC code patterns; seven TACs were unique. In group 1, the most frequently absent teeth were the maxillary lateral incisor of the left side (30%); in group 2, the second premolar of the lower jaw on the right side (9%). Male patients with craniofacial disorder showed a higher percentage of tooth agenesis than female.

**Conclusion:**

The data presented here shows a statistically significant higher prevalence of tooth agenesis in German patients with non-syndromic craniofacial disorder.

**Clinical relevance:**

Radiographic evaluation enables the diagnosis of tooth agenesis. Recognizing early on the higher prevalence of tooth agenesis in patients exhibiting a craniofacial disorder is an important issue when developing long-term and comprehensive interdisciplinary treatment.

## Introduction

The prevalence of cleft lip and/or palate (CL/P) is estimated at about 1 in 600 newborns; it is therefore one of the most frequently occurring craniofacial malformations [1]. The incidence of cleft formation varies according to the geographic location, socioeconomic status, and ethnicity [2, 3], while multifactorial causes, e.g., endogenous and exogenous factors, are also considered as playing a crucial role in CL/P development [2]. Furthermore, there is evidence of a connection between the presence of CL/P and mutations in specific genes [4–8]. An association with some syndromes or sequences, such as Robin sequence (RS), is known. RS is associated with a cleft palate (CP) in 80–90% of the cases [9, 10]. This malformation occurs with the triad of mandibular retrognathia, glossoptosis, and obstructions of the upper airway [11]. The prevalence is 11.3:100,000 live births [12]. Cleft formation is associated with functional disorders including feeding problems and failure to thrive in the first weeks after birth. Other problems include restrictions in oral hygiene, deformations of the dental arch, oronasal fistulas, distinctive skeletal discrepancies between the jaws, and velopharyngeal insufficiencies. All of these lead in turn to hearing and speech problems.

One of the most common dental anomalies is tooth agenesis, also known as congenital tooth absence or hypodontia [13–16]. The permanent dentition is more affected than the primary dentition [17]. Tooth agenesis is caused on a multifactorial level through a number of complex interactions among genetic, environmental, and epigenetic factors during the process of dental development [18]. The occurrence of agenesis differs by sex and geographic location [19]. The prevalence among females is higher than in males. Europeans (5.5%) and Australians showed a higher prevalence than North American populations. The most affected tooth is the second premolar in the mandible (1–5%) followed by the maxillary lateral incisor (0.5–3%), the maxillary second premolar (1–2.5%), and the median mandibular incisor (0.5%). A unilateral occurrence of dental agenesis is more common than a bilateral occurrence [19]. It can appear as a part of a syndrome, such as Van der Woude Syndrome, or in isolated form. However, the frequencies of tooth agenesis increase in combination with the occurrence of a craniofacial disorder [20, 21]. This is due to the close embryological relationship of the occurrence of CL/P and the development of tooth germs in terms of the anatomic position and timing [22]. Causing this combined embryological relationship, there are different genes and gene loci described [23]. CL/P patients have a higher prevalence (31.4 to 50%) of tooth agenesis compared to patients without a craniofacial malformation [24–31]. The number of missing teeth is associated with the extent of the cleft [32, 33]. Variations of tooth shape and structure of both dentitions adjacent to the cleft have been observed [25]. Furthermore, these teeth often show eruption disorders and changes in position. Not only the teeth of the cleft region but also teeth in the posterior region are often not anatomically correct positioned in patients with cleft lip and palate. This mainly affects the second premolar [34]. Due to this, the environmental impact of the surgical closure of the hard palate to be responsible for the loss of these tooth germs is discussed [35]. A milder form of hypodontia involving the asymmetric formation or even the absence of the contralateral teeth is called microform of a CL/P by some authors [36]. Patients with craniofacial disorders need an interdisciplinary rehabilitation therapy that includes neonatologists, cranio-maxillofacial surgeons, otorhinolaryngologists, speech therapists, orthodontists, dentists, and psychologists. The duration and intensity of this therapy depend on the severity of the craniofacial malformation. To set the right time to start the intervention, local data of tooth agenesis and development of the dentition is important, especially for dentists, orthodontists, and cranio-maxillofacial surgeons. Furthermore, enhancing this knowledge about a German population with craniofacial disorder can help to guide health-care professionals in raising awareness of such factors, can help to identify early tooth agenesis, and may give early opportunities to guide and plan the therapy.

The tooth agenesis code (TAC) was designed by van Wink and Tan [37, 38]. It is a common methodology that can provide exact information about the missing teeth, including information about the phenotype of the tooth. It is described in the literature as frequently used for patients with craniofacial disorders [27, 39–42]. Currently, there is limited data available on this topic in the German population.

The aim of this study was to evaluate tooth agenesis using the TAC in German orthodontic patients with non-syndromic craniofacial disorders compared to a healthy control group of patients at the Department of Orthodontics at Tübingen University Hospital, Germany. In addition, our study examined the association of agenesis with the cleft side, skeletal class malformation, sex, craniofacial disorder, and interobserver reliability.

## Materials and methods

### Study design

This cross-sectional study designed to be both retrospective and monocentric was carried out at the Department of Orthodontics at University Hospital Tübingen, Germany. Prior to the start, the study protocol according to the World Medical Association Declaration of Helsinki was approved by the institutional ethics committee of the University hospital Tübingen, Germany (file number: 498/2019BO2).

### Patients

In total, 116 panoramic radiographs of the Department of Orthodontics at the University Hospital Tübingen, Germany, were analyzed for this study. The data were obtained from the records of patients at the evaluation in the Department of Orthodontics at the University Hospital Tübingen, Germany. The sample size was calculated with a two-sided two-sample *t*-test method by a statistician during the study planning. The composition of the sample size for the group of patients with craniofacial disorder is oriented to the current patient cases of the Department of Orthodontics at the University Hospital Tübingen, Germany. The timeline of participants’ recruitment was from April to October 2019. It consisted of Caucasian male and female patients.

The following inclusion criteria were used for recruitment:

- Current orthodontic treatment in our department.
- Age between 5 and 18 years. Patients younger than 5 years were excluded, because the tooth development does not allow identifying tooth agenesis in a radiograph at this age [43, 44]. And especially patients with a CL/P show a statistically significant delay of mineralization of the second premolars [45, 46]. Also, patients older than 18 years were excluded, due to the fact that the tooth development and facial growth of patients older than 18 years should be completed [47].
- Non-syndromic craniofacial malformations such as CL/P and RS. The diagnosis was confirmed by the neonatal picture or pre-operative record.
- Patients with a panoramic radiograph and a lateral cephalograms according to the ALARA (as low as reasonably achievable) principles with an indication justifying radiation exposure within the course of orthodontic treatment.
  The exclusion criteria were defined as follows:
- Additional associated complex congenital malformations (syndromes) or mental retardation, due to the fact that some syndromes are associated with tooth agenesis [48, 49].
- Patients younger than 5 years and older than 18 years.
- Radiographs of insufficient quality for diagnostic purposes (e.g., overexposure).

The patients were divided into two groups:

- Group one — patients with craniofacial disorders, i.e., CL/P and RS
- Group two — patients without craniofacial disorder; healthy control group.

The inclusion criteria for group 1 represented the presence of a non-syndromic craniofacial malformation, e.g., at least a cleft of the soft palate. Therefore, this group is composed of patients with CL/P and RS. All types of clefts were included in this study, including patients with a Simonart’s band with and without a hard tissue bridge. Group 1 was compared with a healthy control group (group two) containing patients without craniofacial disorder.

### Instruments for dental assessment

The panoramic radiographs were scored for tooth agenesis using the TAC by an experienced examiner at the Department of Orthodontics. The TAC is a binary system with “zero” coding the presence of the tooth. The values of a missing tooth are shown in Table 1 [37, 38]. The values in line B are associated with the missing teeth in the respective quadrant. A certain quadrant without tooth agenesis would have the TAC value of 000. According to the TAC system, a quadrant with all missing teeth excluding the wisdom teeth has the value of 127. The TAC code of the whole dentition has twelve numbers, three numbers for each quadrant. For example, a TAC code of 016 018 000 000: 016 corresponds to the first quadrant with a missing second premolar, 018 to the second quadrant with an agenesis of the lateral incisor and the second premolar, 000 to the third quadrant, and 000 to the fourth quadrant with no missing teeth. The presence of wisdom teeth was not included in this study. This is due to the fact of the inaccuracy of identifying tooth agenesis at the chosen minimum age of 5 years, because the natural development of these tooth germs begins at the age of 5 till 9 years and to the comparability to other studies [50].

**Table 1:**
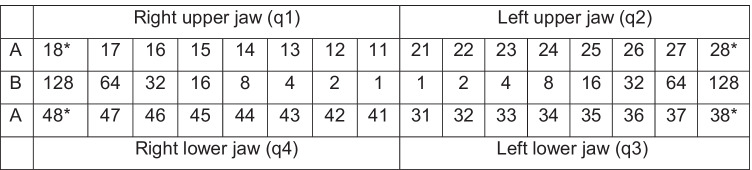
Schematic representation of the binary arithmetic system of tooth agenesis code (TAC) assigning unique values to determine dental agenesis

A: schematic representation of tooth numbering according to the Fédération Dentaire Internationale (FDI) system [48]. B: binary arithmetic system of TAC (first quadrant (q1), second quadrant (q2), third quadrant (q3), fourth quadrant (q4))

^*^Presence of wisdom teeth was not included in this study

### Reliability measurement

To assess the interobserver reliability, the panoramic radiographs were evaluated by two experienced examiners (ML, CW) applying the TAC. For this purpose, one of these examiners (ML) assessed the total (*n* = 116) of panoramic radiographs and examined a part of radiographs (*n* = 37) twice. The second examiner (CW) evaluated the 37 radiographs independently of the first examiner, spatially and temporally separated.

### Statistical data analyses

The patient data were collected out of the department’s electronic database with clinical records and saved pseudonymized in an Excel® sheet (Microsoft Inc., Redmond, Washington, USA). Statistical evaluation, descriptive statistics, and analysis were performed using JMP (Version 15.2.0, SAS Institute Inc., Cary, USA). Test–retest reliability was determined with the Cohen’s kappa. Pearson’s *χ*^2^ statistics were applied to examine the association of agenesis with craniofacial disorder and against the control group. In addition, other variables, such as craniofacial disorder, cleft side, the distribution of skeletal class, and patient sex, were statistically analyzed. The significance level was set up at 5% (*α* = 0.05).

### Skeletal class malformation

The sagittal relationship of maxilla and mandible, called skeletal class, was determined via lateral cephalograms using the ANB angle according to individualized cephalometric of Hasund analysis. This is not a statistical evaluation. It is a necessary and routine part of an orthodontic diagnosis based on the guidelines of the current scientific literature to ensure a high quality of care. The skeletal class data were collected of the department’s electronic database with routine clinical records.

## Results

### Characteristics of patients

Out of 116 patients, 108 met the inclusion criteria for this study. The eight patients who were excluded were younger than 5 years. These patients were only used for the reliability measurement of the two examiners (ML and CW).

The characteristics and distribution of the patients included in this study are shown in Table 2. The records of 108 patients (48.15% male and 51.85% female) were examined. The 108 patients were divided into the craniofacial disorder group 1 (*n* = 43) and the healthy control group 2 (*n* = 65). The age distribution ranged from 5 years and 4 months to 17 years and 1 month; the average age of the patients at the time of the x-ray diagnosis was 9 years and 9 months ± 2 years and 6 months regarding both groups together. The average age was 8.88 years for group 1 and 10.80 years for group 2. Group 1 consisted of 60.46% male patients and 39.53% female. Group 2 had 40.00% male patients and 60.00% female. Regarding the skeletal class, group 1 showed configuration III in 69.77% of the cases as the most frequent and group 2 class II in 44.62% of the cases. The craniofacial disorder distribution of group 1 showed that the most frequent malformation was the unilateral cleft on the left side with 24 patients followed by the bilateral cleft formation with eight patients. Three patients with Robin sequence and five patients with cleft palate participated in this study.

**Table 2:** Characteristics and distribution of patients in groups 1 and 2

*n* = 108		Group 1 (*n* = 43)		Group 2 (*n* = 65)
	*n*	%	*n*	%
Age				
Mean	8.88		10.80	
SD	1.92		2.71	
Sex				
Male	26	60.46	26	40.00
Female	17	39.53	39	60.00
Skeletal class				
Class I	3	6.98	14	21.54
Class II	10	23.26	29	44.62
Class III	30	69.77	22	33.85
Craniofacial disorder				
RS	3	6.98	-	-
CLP	35	81.40	-	-
CP	5	11.63		
Cleft location				
Unilateral	27		-	-
Left	24		-	-
Right	3		-	-
Bilateral	8		-	-

*SD* standard deviation, *CLP* cleft lip and palate, *CP* cleft palate, *RS* Robin sequence, *m* male, *f* female

### Reliability measurements

The interrater reliability measurement with the Cohen’s kappa showed a value of 0.52. The degree of agreement between the two observers was thus moderate, and the match corresponds to the expected random match. The assessment results should be as independent as possible from the respective assessor and ideally even identical. However, this cannot be assumed in reality due to systematic and random deviations errors of each observer.

### General analysis of tooth agenesis code in groups 1 and 2

Table 3 shows a complete and detailed overview of the prevalence of the TAC values according to tooth type of groups 1 and 2. In group 1, tooth agenesis was found in 19 cases (44.19%) of all 43 patients. The teeth of the upper jaw were the most frequently absent teeth, particularly the lateral incisors (22 = 30.23%) of the second quadrant, followed by the lateral incisor (12 = 13.95%) and the second premolar (15 = 13.95%) located in the first quadrant. The second premolar of the second quadrant showed an agenesis in five cases (11.63%), followed by the second premolar of the fourth quadrant with a percentage of 9.30%. Regarding group 2, an agenesis was found in 9 cases (13.85%). The most frequently absent teeth in group 2 were the lateral incisors of the first quadrant (12 = 4.62%), the median incisor and the second premolar of the fourth quadrant (41, 45 = 4.62%), and the second premolar of the third quadrant (35 = 4.62%). In the upper jaw, the second premolar of the first quadrant and lateral incisor of the second quadrant showed the same percentage values (15, 22 = 3.08%). In the upper jaw, the first premolar of the first quadrant and the median incisor and the first premolar of the second quadrant showed the same percentage values (14, 21, 24, 31, 37 = 1.54%). Regarding the lower jaw, the median incisor and the second molar of the third quadrant had the same prevalence of tooth absence (31, 37 = 1.54%).

**Table 3:**
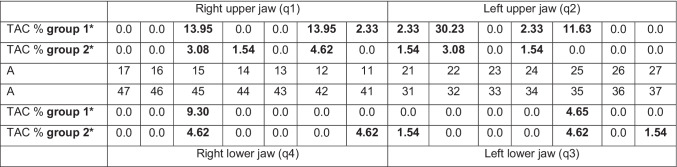
Prevalence of absence per tooth (percentage) in 108 patients of groups 1 and 2

A: schematic representation of tooth numbering according to the Fédération Dentaire Internationale (FDI) system [48]

*TAC %* percentage TAC values according to tooth type

^*^Presence of wisdom teeth was not included

Table 4 shows the distribution of the TAC of groups 1 and 2. In group 1, there were 14 different possibilities of TAC codes. A total of ten codes were unique, meaning that this code was only observed in one patient within the entire group 1. The distribution of the TAC codes in group 2 showed nine different possibilities of TAC code patterns. Seven TACs were unique in this group.

**Table 4:** Tooth agenesis code (TAC) of groups 1 and 2, frequency and missing teeth in the entire mouth

		Frequency *N*	Teeth missing			
	Total		Maxilla		Mandibular	
			Left	Right	Left	Right
Group 1						
1	000 000 000 000	24				
2	000 002 000 000	5	22			
3	000 002 000 016	1	22			45
4	000 002 016 016	1	22		35	45
5	000 016 000 000	1	25			
6	000 018 000 000	1	22, 25			
7	002 000 000 000	1		12		
8	002 010 000 000	1	22, 24	12		
9	002 016 000 016	1	25	12		45
10	002 018 000 000	2	22	12, 15		
11	003 003 000 000	1	21, 22	11, 12		
12	016 000 000 000	2		15		
13	016 016 016 016	1	25	15	35	45
14	016 018 000 000	1	22, 25	15		
Group 2						
1	000 000 000 000	56				
2	000 000 000 001	2				41
3	000 000 064 000	1			37	
4	000 001 000 000	1	21			45
5	000 002 000 016	1	22			45
6	002 000 016 000	1		12	35	
7	010 010 000 000	1	22, 24	12, 14		
8	016 000 016 016	1		15	35	45
9	018 000 017 001	1		12, 15	31, 35	41

### Relationship of agenesis between patient sex, skeletal class, and craniofacial disorder

Table 5 shows the results of the descriptive analysis regarding the prevalence of tooth agenesis in relation to sex, skeletal class, type of craniofacial malformation, and type of cleft of groups 1 and 2. The prevalence of agenesis was 44.19% in group 1 and 13.85% in group 2, respectively. This contribution was statistically significant (*p* = 0.0162; *χ*^2^). The prevalence of agenesis of the different craniofacial disorders in RS patients obtained an average of 0.00%, in patients with a left-sided CLP 30.23% and right-sided 2.33%, bilateral 6.98%, and CP 4.65%. According to the patient’s sex in group 1, higher percentages of tooth agenesis were found in male patients, with males at 25.58% and females at 18.60%, but this was not statistically significant. In group 2, no difference was found in both groups analyzing the sex. Regarding the localization of the agenesis in group 1, the distribution showed that 20.03% were isolated in the upper jaw and 9.30% in the lower and in the upper jaw. No tooth agenesis was found isolated in the lower jaw. These results were statistically significant (*p* < 0.0001 (*χ*^2^)). Group 2 had a distribution of 1.54% in the upper jaw, 3.08% in the lower, and 7.69% mixed in the lower and upper jaw together. These results were also statistically significant (*p* < 0.0001 (*χ*^2^)). The descriptive analysis of the skeletal class malformation showed no statistical significance in both groups. Skeletal class III had the highest percentage of tooth agenesis in group 1 with 34.88%. In group 2, skeletal class II showed the highest value of tooth agenesis with 7.69%. Regarding patients with unilateral CLP, the prevalence of agenesis was higher in the cleft side compared to non-cleft side in the maxilla. However, this was not statistically significant.

**Table 5:** Percentage and relative frequency using Pearson’s *χ*^2^ test to assess an association between tooth agenesis and the other variables (skeletal class malformation, craniofacial malformation, cleft type, and sex)

*n* = 108	Group 1 (*n* = 43)	Group 2 (*n* = 65)
Tooth agenesis *p value* = *0.0162 (χ*^*2*^*)*	44.19%	13.85%
Sex		
Male	25.58%	7.69%
Female	18.60%	7.69%
*p value*	*0.7590 (χ*^*2*^*)*	*0.4829 (χ*^*2*^*)*
Skeletal class		
Class I	0.00%	1.54%
Class II	9.30%	7.69%
Class III	34.88%	6.15%
*p value*	*0.2396 (χ*^*2*^*)*	*0.6251 (χ*^*2*^*)*
Craniofacial disorder		
RS	0.00%	
Unilateral CLP		
Left	30.23%	
Right	2.33%	
Bilateral	6.98%	
CP	4.65%	
*p value*	*0.4528 (χ*^*2*^*)*	
Upper/lower jaw		
Maxilla	20.93%	1.54%
Mandible	0.00%	3.08%
Maxilla and mandible	9.30%	7.69%
*p value*	< *0.0001 (χ*^*2*^*)*	< *0.0001 (χ*^*2*^*)*

Significance level = 0.05

*CLP* cleft lip and palate, *CP* cleft palate, *RS* Robin sequence

## Discussion

The aim of this study was to evaluate tooth agenesis using the tooth agenesis code (TAC) method in German orthodontic patients with non-syndromic CL/P and RS compared to a healthy control group in the patients of the Department of Orthodontics of the Tübingen University Hospital in Germany. This is a novel approach regarding the cohort group of patients with CL/P and RS. There was a statistical significance in the homogeneity distribution between the tooth agenesis in both groups. The craniofacial disorder group showed a higher prevalence in tooth agenesis than the control group. Dental agenesis has been described as a frequently occurring anomaly among patients with CL/P and RS [33, 40, 51–55]. In the current literature, there is an association between the cleft side and the side of the hypodontia. Bartzela et al. showed in a study that the prevalence of orofacial cleft and cleft side is more frequent on the left side. This is due to the fact that the localization of a cleft is more frequently present on the left side [40]. This finding was confirmed by the present study, although it was not statistically significant. Regarding the TAC distribution of absent teeth in the upper and lower jaw, the upper jaw reached a statistically significant higher prevalence in the craniofacial disorder group compared to the healthy control group, especially considering the lateral incisors (30.23%) in the second quadrant and the second premolar (13.95%) in the first quadrant. The results of the present study can only be compared to a few studies using the TAC binary code system without examining the wisdom teeth. Findings in the literature agree with those in the current study, indicating that the most frequent absent tooth was the maxillary lateral incisor and the mandibular second premolar [27, 40]. Patients with RS had an agenesis of 0%. This result does not support recent findings in the literature. This might be due to the small sample size of RS patients in our study. The tooth agenesis of RS patients in a recent meta-analysis showed values between 42 and 47.8% and is more prevalent in RS than in CP patients overall [53, 55–57].

Fourteen different TAC patterns were assessed in the craniofacial disorder group 1. A total of ten patterns occurred only once. In a study of López-Giménez et al., a total of nineteen different tooth agenesis codes were found in patients with uni- and bilateral CL/P [27]. The comparison of the TAC patterns with other studies is difficult because of differences in the composition of the craniofacial anomalies in the study groups [51, 58].

Regarding the prevalence of hypodontia in the general population with percentages between 2.3 and 7.6%, the healthy control group of this study had a higher prevalence, with 13.85% [19]. Celikoglou et al. found the prevalence of tooth agenesis at 4.3% in Turkish orthodontic patients [59]. The high prevalence could be due to the fact that these patients are in orthodontic treatment, and one possible cause of this treatment could be agenesis [59, 60]. This fact was not considered in the inclusion criteria of this study. Nonetheless, the findings of the agenesis of the phenotype in this study of the healthy control group is consistent with the current literature [19].

The prevalence of tooth agenesis in skeletal class III malocclusion of the craniofacial disorder group showed the highest value with 34.88% followed by class II with 9.30%. The control group had the highest value in skeletal class II with a prevalence of 7.96%, but directly followed by class III malocclusion with 6.15%. Different conclusions were found in the literature regarding this topic. Celikoglu et al. demonstrated in a study that tooth agenesis was statistically significantly lower in orthodontic patients without craniofacial disorder showing a skeletal class II malformation. Costa et al. showed that the most prevalent skeletal malocclusion in non-syndromic orthodontic patients was class I, followed by class II and then class III [61].

In this study, male patients in the group with a craniofacial malformation showed a higher prevalence in tooth agenesis than female patients, though our findings here are not statistically significant. The percentage of the healthy control group is balanced in both sexes. The same result was shown in a study with Brazilian non-syndromic orthodontic patients [61]. But this is not similar to the recent population examined in a meta-analysis of 2004. Females showed a 1.37 times higher prevalence in missing teeth than males [19].

The age range of the patients analyzed in this study can be regarded as representative of general experience in orthodontic treatment, depending on the typical starting time for orthodontic therapy in children, usually in the second phase of exfoliation. The starting time of the orthodontic treatment for patients with craniofacial disorders is earlier, due to the need for more comprehensive treatment and for longer periods of therapy. Major skeletal discrepancies in these patients, as well as the younger age distribution of the craniofacial disorder group, require alternatives in orthodontic treatment practices.

The TAC system for evaluating and characterizing tooth agenesis by using a unique code for each quadrant was a useful method and easy to use for panoramic radiographs. The two examiners in this study found the method easy to implement in everyday clinical routine for the examination of radiographs, and for use not only in patients with craniofacial disorders but also in those receiving general orthodontic treatment in our clinic. The interobserver kappa showed a value of 0.52. The match corresponds to the expected random match, and the degree of agreement between the two observers of the radiographs was moderate. The kappa value does not indicate a high correlation between different examiners in this study. A source of bias could be the small sample size in this study. Furthermore, it could be due to the fact that radiography may not completely fulfill the criteria of good practice, so the examiner could interpret the radiography different. The observer experience should not play a role, since both had a similar level of expertise at the time of the examination and the same amount of work experience. Nonetheless, the result confirmed the TAC system as a useful method for scoring panoramic radiographs. Furthermore, it can be used in other dental areas where tooth agenesis is important for planning treatment [37, 38]. The advantage of this system is not only the assessment of the absent tooth, but the definition of the phenotype to the corresponding quadrant. This method makes it easy, especially in patients with CL/P or other disorders of the jaw to perform an exact assignment to the localization of the malformation and, thus, to establish an association. The knowledge about a higher prevalence of tooth agenesis in these challenging patients leads to an improved interdisciplinary treatment planning.

### Limitations and outlook

One limitation of this study is the unequal sample size of the groups depending on the recruitment of the patient’s data. In a future study, a larger and equal sample size is needed especially for the RS group. This study would be interesting to be multicentric in Germany, like for example the study of Bartzela et al., who evaluated radiographs of three Cleft Palate Centers [40]. However, patient recruitment will remain difficult as it depends on the prevalence of each craniofacial malformation. Furthermore, the TAC of the third molar could be included in future studies to determine a relationship between agenesis of the wisdom teeth, the remaining teeth in the dentition and, in addition, to the craniofacial disorder.

In a follow-up study, the angle of the inclination position and the location of the sector of the canine in the maxilla were evaluated in patients according to the same criteria as applied in the study presented here. This provided information about the displacement tendency of the canines, allowing orthodontic treatment to be adapted to the situation.

## Conclusion

The data presented here showed a statistically significant higher prevalence of tooth agenesis in orthodontic patients with a non-syndromic craniofacial disorder than in patients without a craniofacial disorder. Eighteen different TAC code patterns were assessed in the group with craniofacial malformation and a total of fourteen were unique. The lateral incisors of the upper jaw were the most frequently absent tooth in this group. Male patients with craniofacial disorder showed a higher percentage of tooth agenesis than female. No tooth agenesis was found isolated in the lower jaw of patients with craniofacial disorder. The higher prevalence of tooth agenesis in patients with a craniofacial disorder is an important issue to consider when developing long-lasting and comprehensive interdisciplinary therapies.

## Abbreviations

BCLP: Bilateral cleft lip and palate
CL/P: Cleft lip and/or palate
CLP: Cleft lip and palate
CP: Cleft palate
RS: Robin sequence
TAC: Tooth agenesis code
UCLP: Unilateral cleft lip and palate

## References

[1] Cobourne MT (2004). The complex genetics of cleft lip and palate. Eur J Orthod.

[2] Murray JC (2002). Gene/environment causes of cleft lip and/or palate. Clin Genet.

[3] Cox TC (2004). Taking it to the max: The genetic and developmental mechanisms coordinating midfacial morphogenesis and dysmorphology. Clin Genet.

[4] Ardinger HH, Buetow KH, Bell GI (1989). Association of genetic variation of the transforming growth factor-alpha gene with cleft lip and palate. Am J Hum Genet.

[5] Romitti PA, Lidral AC, Munger RG (1999). Candidate genes for nonsyndromic cleft lip and palate and maternal cigarette smoking and alcohol consumption: evaluation of genotype-environment interactions from a population-based case-control study of orofacial clefts. Teratology.

[6] Zucchero TM, Cooper ME, Maher BS (2004). Interferon regulatory factor 6 (IRF6) gene variants and the risk of isolated cleft lip or palate. N Engl J Med.

[7] Avila JR, Jezewski PA, Vieira AR (2006). PVRL1 variants contribute to non-syndromic cleft lip and palate in multiple populations. Am J Med Genet.

[8] van den Boogaard MJ, Dorland M, Beemer FA (2000). MSX1 mutation is associated with orofacial clefting and tooth agenesis in humans. Nat Genet.

[9] Marques IL, de Sousa TV, Carneiro AF (2005). Seqüência de Robin: protocolo único de tratamento. J Pediatr (Rio J).

[10] Caouette-Laberge L, Bayet B, Larocque Y (1994). The Pierre Robin sequence: review of 125 cases and evolution of treatment modalities. Plast Reconstr Surg.

[11] Robin P (1994) A fall of the base of the tongue considered as a new cause of nasopharyngeal respiratory impairment: Pierre Robin sequence, a translation. 1923. Plast Reconstr Surg 93(93):1301–1303 8171154

[12] Maas C, Poets CF (2014). Initial treatment and early weight gain of children with Robin Sequence in Germany: a prospective epidemiological study. Arch Dis Child Fetal Neonatal Ed.

[13] Jorgenson RJ (1980). Clinician's view of hypodontia. J Am Dent Assoc.

[14] Vastardis H (2000). The genetics of human tooth agenesis: new discoveries for understanding dental anomalies. Am J Orthod Dentofac Orthop.

[15] Nunn JH, Carter NE, Gillgrass TJ (2003). The interdisciplinary management of hypodontia: background and role of paediatric dentistry. Br Dent J.

[16] de Coster PJ, Marks La, Martens LC (2009). Dental agenesis: genetic and clinical perspectives. J Oral Pathol Med.

[17] Cobourne MT (2007). Familial human hypodontia–is it all in the genes?. Br Dent J.

[18] Brook AH, Griffin RC, Smith RN (2009). Tooth size patterns in patients with hypodontia and supernumerary teeth. Arch Oral Biol.

[19] Polder BJ, Van’t Hof MA, van der Linden FP (2004). A meta-analysis of the prevalence of dental agenesis of permanent teeth. Commun Dent Oral Epidemiol.

[20] Khalaf K, Miskelly J, Voge E (2014). Prevalence of hypodontia and associated factors: a systematic review and meta-analysis. J Orthod.

[21] Ranta R (1984). Associations of some variables to tooth formation in children with isolated cleft palate. Eur J Oral Sci.

[22] Stahl F, Grabowski R, Wigger K (2006). Epidemiology of Hoffmeister's "genetically determined predisposition to disturbed development of the dentition" in patients with cleft lip and palate. Cleft Palate-Craniofacial J.

[23] Phan M, Conte F, Khandelwal KD (2016). Tooth agenesis and orofacial clefting: genetic brothers in arms?. Hum Genet.

[24] Ranta R (1983). Hypodontia and delayed development of the second premolars in cleft palate children. Eur J Orthod.

[25] Ranta R (1986). A review of tooth formation in children with cleft lip/palate. Am J Orthod Dentofac Orthop.

[26] Larson M, Hellquist R, Jakobsson OP (1998). Dental abnormalities and ectopic eruption in patients with isolated cleft palate. Scand J Plast Reconstr Surg Hand Surg.

[27] López-Giménez A, Silvestre-Rangil J, Silvestre FJ (2018). Tooth agenesis code (TAC) in complete unilateral and bilateral cleft lip and palate patients. Odontology.

[28] de Lima Pedro R, Daniel Brito Faria M, de Castro Costa M (2012). Dental anomalies in children born with clefts: a case-control study. Cleft Palate-Craniofacial J.

[29] Olin WH (1964). Dental anomalies in cleft lip and palate patients. The Angle Orthod.

[30] Kraus BS, Jordan RE, Pruzansky S (1966). Dental abnormalities in the deciduous and permanent dentitions of individuals with cleft lip and palate. J Dent Res.

[31] Slayton RL, Williams L, Murray JC (2003). Genetic association studies of cleft lip and/or palate with hypodontia outside the cleft region. Cleft Palate-Craniofacial J.

[32] Haring FN (1976). Dental development in cleft and noncleft subjects. Angle Orthod.

[33] Möller LH, Pradel W, Gedrange T (2021). Prevalence of hypodontia and supernumerary teeth in a German cleft lip with/without palate population. BMC Oral Health.

[34] Opitz C (2002). Kieferorthopädische Behandlung von Patienten mit Lippen-Kiefer-Gaumen-Spalten.

[35] Lekkas C, Latief BS, ter Rahe SP (2000). The adult unoperated cleft patient: absence of maxillary teeth outside the cleft area. Cleft Palate-Craniofacial J.

[36] Fukuhara T, Saito S (1963) Possible carrier status of hereditary cleft palate with cleft lip: report of cases. Bull Tokyo Dent Coll 10:333–345

[37] van Wijk AJ, Tan SPK (2006). A numeric code for identifying patterns of human tooth agenesis: a new approach. Eur J Oral Sci.

[38] Tan SPK, van Wijk AJ, Prahl-Andersen B (2011). Severe hypodontia: identifying patterns of human tooth agenesis. Eur J Orthod.

[39] Hermus RR, van Wijk AJ, Tan SPK (2013). Patterns of tooth agenesis in patients with orofacial clefts. Eur J Oral Sci.

[40] Bartzela TN, Carels CEL, Bronkhorst EM (2010). Tooth agenesis patterns in bilateral cleft lip and palate. Eur J Oral Sci.

[41] Bartzela TN, Carels CEL, Bronkhorst EM (2013). Tooth agenesis patterns in unilateral cleft lip and palate in humans. Arch Oral Biol.

[42] Zhu J, Zheng S, Ge L (2013). Analysis of dental agenesis patterns of the oligodontia patients using the method of tooth agenesis code. Zhonghua Kou Qiang Yi Xue Za Zhi.

[43] Ravin JJ, Nielsen HG (1977). A longitudinal radiographic study of the mineralization of 2nd premolars. Scand J Dent Res.

[44] Nielsen HG, Ravn JJ (1976). A radiographic study of mineralization of permanent teeth in a group of children aged 3–7 years. Scand J Dent Res.

[45] Mitsea AG, Spyropoulos MN (2001). Premolar development in Greek children with cleft lip and palate. Quintessence Int.

[46] Borodkin AF, Feigal RJ, Beiraghi S (2008). Permanent tooth development in children with cleft lip and palate. Pediatr Dent.

[47] Susanne C, Guidotti A, Hauspie R (1985) Age changes of skull dimenions. Anthropol Anz 43(1):31–6 3994331

[48] de Santis D, Sinigaglia S, Faccioni P (2019). Syndromes associated with dental agenesis. Minerva Stomatol.

[49] Stavropoulos D, Bartzela T, Bronkhorst E (2011). Dental agenesis patterns of permanent teeth in Apert syndrome. Eur J Oral Sci.

[50] Logan WHG, Kronfeld R (1933). Development of the human jaws and surrounding structures from birth to the age of fifteen years**from the Research Department of the Chicago College of Dental Surgery, Dental Department of Loyola University. Read at the Third General Meeting of the Seventy-Fourth Annual Session of the American Dental Association, Buffalo, N. Y., Sept. 14, 1932. J Am Dent Assoc.

[51] Fishman LS (1970). Factors related to tooth number, eruption time, and tooth position in cleft palate individuals. ASDC J Dent Child.

[52] Menezes R, Vieira AR (2008). Dental anomalies as part of the cleft spectrum. Cleft Palate-Craniofacial J.

[53] Antonarakis GS, Suri S (2014). Prevalence and patterns of permanent tooth agenesis in patients with nonsyndromic Pierre Robin sequence. Am J Orthod Dentofac Orthop.

[54] Tannure PN, Oliveira CAGR, Maia LC (2012). Prevalence of dental anomalies in nonsyndromic individuals with cleft lip and palate: a systematic review and meta-analysis. Cleft Palate-Craniofacial J.

[55] Dillon M, Seshu M, Flannigan N et al (2021) How does hypodontia compare in nonsyndromic Pierre Robin sequence versus isolated cleft palate and isolated cleft lip? Cleft Palate Craniofac J 59(5):603–608. 10.1177/10556656211017778 10.1177/10556656211017778 34018409

[56] de Smalen A, van Nunen DPF, Hermus RR (2017). Permanent tooth agenesis in non-syndromic Robin sequence and cleft palate: prevalence and patterns. Clin Oral Investig.

[57] Andersson Els-Marie, Feragen Kristin Billaud, Mikalsen Daniel (2015). Bilateral hypodontia in adolescents with Pierre Robin sequence. Cleft Palate-Craniofacial J.

[58] Peck S, Peck L (1996). Tooth numbering progress. Angle Orthod.

[59] Celikoglu M, Kazanci F, Miloglu O (2010). Frequency and characteristics of tooth agenesis among an orthodontic patient population. Med Oral Patol Oral Cir Bucal.

[60] Celikoglu M, Buyuk SK, Sekerci AE (2015). Maxillary dental anomalies in patients with cleft lip and palate: a cone beam computed tomography study. J Clin Pediatr Dent.

[61] Costa AMG, Trevizan M, Matsumoto MAN (2017). Association between tooth agenesis and skeletal malocclusions. J Oral Maxillofac Res.

